# Clinical characteristics and prognosis of childhood-onset lupus mesenteric vasculitis as the initial presentation—a case–control study

**DOI:** 10.1186/s13075-023-03237-x

**Published:** 2023-12-20

**Authors:** Jia Zhu, Jianming Lai, Xiaohui Liu, Xue Zhao, Ran Tao, Min Kang, Xiaolan Huang, Li Wang, Fengqi Wu, Xiaoping Pan, Gaixiu Su

**Affiliations:** 1 Department of Rheumatology and Immunology, Children’s Hospital, Capital Institute of Pediatrics. , NO.2 Yabao Road, Chaoyang District, Beijing, 100020 China; 2 Department of Rheumatology and Immunology, Jiangxi Provincial Children’s Hospital, No. 1666, Diezihu Avenue, Honggutan District, Nanchang, 330013 Jiangxi Province China; 3 Department of Pediatric, The Second Hospital of Hebei Medical University, No. 215, Heping West Road, Xinhua District, Shijiazhuang, 050000 Hebei Province China; 4 Department of Radiology, Children’s Hospital, Capital Institute of Pediatrics, NO.2 Yabao Road, Chaoyang District, Beijing, 100020 China; 5 Experimental Research Center, Capital Institute of Pediatrics, NO.2 Yabao Road, Chaoyang District, Beijing, 100020 China; 6 Capital Institute of Pediatrics, NO.2 Yabao Road, Chaoyang District, Beijing, 100020 China; 7 Department of Information Management, The National Center for Women’s and Children’s Health of the Chinese Center for Disease Control and Prevention, NO.27 Nanwei Road, Xicheng District, Beijing, 100050 China

**Keywords:** Childhood-onset systemic lupus erythematosus, Lupus mesenteric vasculitis, Clinical characteristics, Prognosis

## Abstract

**Background:**

Lupus mesenteric vasculitis (LMV) as initial presentation is rare, especially in childhood-onset systemic lupus erythematosus (cSLE). It is a critical complication of lupus. At present, the research on cSLE with LMV as the initial presentation is few. The aim of this study was to analyze the clinical characteristics and prognosis of cSLE with LMV in the Chinese population, compared with non-LMV cSLE.

**Methods:**

A retrospective case-controlled study was conducted on 55 cSLE patients between July 2018 and July 2021. The clinical data, laboratory findings, imaging, treatment, and follow-up data were collected and compared between the two groups of cSLE with LMV and non-LMV. Non-LMV cSLE patients were matched according to the age and sex of LMV patients.

**Results:**

A total of 11 cSLE patients with LMV as the LMV group and 44 cSLE patients without LMV as the non-LMV group were included. The average age of onset was 12.55 ± 1.57 years old, the male-to-female ratio was 2:9, and high disease activity was observed in the LMV group. Abdominal pain was most common in LMV. Compared with the non-LMV, the percentage of abdominal pain, vomiting, abdominal distension, and diarrhea was higher, and gastrointestinal tract, serous cavity, kidney, and lung damage were higher in the LMV group (*P* < 0.05). In abdominal-enhanced CT, the percentage of intestinal wall thickening, peritoneal effusion, mesenteric vascular enhancement, hydronephrosis with ureteral dilatation, intestinal congestion, and gastric mucosa thickening in the LMV group were higher than those in the non-LMV group (*P* < 0.05). The percentage of receiving methylprednisolone pulse combined with cyclophosphamide pulse therapy in LMV was higher than in non-LMV. The clinical symptoms disappeared quickly, and there were no deaths in the LMV group. Compared with the non-LMV group, the 24-h urinary protein was higher, the complement C3 was lower, and the disease activity was higher in the LMV group (*P* < 0.05).

**Conclusions:**

LMV often occurs in 12 ~ 13-year-old girls with high disease activity of cSLE. Abdominal pain is the most common and more susceptible to damage to the kidney, serous cavity, and lung in cSLE with LMV. Methylprednisolone pulse combined with CTX pulse therapy is effective. After the treatment above, cSLE with LMV has a good prognosis, but the overall recovery is worse than non-LMV patients.

## Background

Childhood-onset systemic lupus erythematosus (cSLE) refers to systemic lupus erythematosus (SLE) that occurs in childhood, which is a chronic autoimmune disease with unknown causes. Systemic lupus erythematosus can affect almost all organs. The gastrointestinal system is relatively common, with about 40% of SLE patients involved, but lupus mesenteric vasculitis (LMV) is rare. The literature reports that the prevalence of LMV is 0.2 to 9.7% [1]. LMV as initial presentation is rarer, especially in cSLE. At present, the etiology of LMV is unknown, and the possible causes include the formation of an immune complex in the mesenteric vascular wall, thrombosis, etc. LMV can cause intestinal wall edema, ulcer, hemorrhage, necrosis, and even perforation [1]. It is a critical complication of SLE, which requires clinicians to be highly alert and timely identify. At present, the research on cSLE with LMV as the initial presentation is few. We summarized and analyzed these cases to explore the clinical characteristics and prognosis of cSLE with LMV.

## Methods

### Study design

This was a retrospective case–control study. We collected the medical record, laboratory findings, imaging, treatment data of the first hospitalization, and follow-up materials including 1 month, 3 months, 6 months, and 12 months after the initial treatment. The LMV group: the cSLE with LMV as initial presentation who were diagnosed for the first time in the Department of Rheumatology and Immunology, Children’s Hospital, Capital Institute of Pediatrics, the Second Hospital of Hebei Medical University, and Jiangxi Provincial Children’s Hospital from July 2018 to July 2021. The non-LMV group: the cSLE without LMV at onset, who were diagnosed for the first time in the Department of Rheumatology and Immunology, Children’s Hospital, Capital Institute of Pediatrics, during the same period. We selected non-LMV cases who matched according to the age and sex of the LMV group, and the ratio of the LMV group to the non-LMV group = 1:4. All patients were ethnic Chinese. Exclusion criteria included the following diseases: (1) infection condition: exclusion through the following tests—stool routine, stool culture, serum Epstein-Barr virus deoxyribonucleic acid, serum antibodies of coxsackie virus, enteric cytopathic human orphan virus, mycoplasma pneumonia, and so on; (2) tumor; (3) genetic metabolic disease; (4) previous intestinal disease, such as chronic diarrheal disease, inflammatory bowel disease, and so on; (5) previous other chronic diseases, such as hypertension, diabetes, etc. The diagnosis of cSLE must comply with the 2019 European League against Rheumatology/American College of Rheumatology (EULAR/ACR) SLE diagnostic criteria [2], and the age is less than 18 years old. LMV was diagnosed by abdominal-enhanced CT, showing at least three or more of the following imaging: intestinal wall thickening, “target line sign,” segmental intestinal dilatation, mesenteric vascular enhancement, and mesenteric fat blurring [3]. Organ damage was determined according to 2019 EULAR/ACR SLE diagnostic criteria. To determine the disease activity of cSLE, Systemic Lupus Erythematosus Disease Activity Index-2000 (SLEDAI-2 K) was used: a score of less than 4 points indicates that the disease is inactive, more than 5 points indicate that the disease is active, and the higher the score, the higher disease activity. This study was approved by the ethics committee of the Capital Institute of Pediatrics.

### Statistical analysis

Categorical variables are presented as the number of cases and frequencies (percentages), while continuous variables are reported as means ± standard deviation. When comparing the two groups, if it is a categorical variable, *χ*^2^or ${\chi }_{{\text{C}}}^{2}$ inspection or Fisher’s exact inspection is adopted; if it is a continuous variable, ANOVA or Student’s *t* test was used. Levene’s test was used to compare the overall variance of the two groups (*α* = 0.10); if the overall variance is not uniform, the *t* test is adopted. Set inspection level *α* = 0.05. Statistical analyses were performed using IBM SPSS Statistics (version 15.0).

## Results

### Basic information

A total of 11 cSLE with LMV were included in the LMV group, and the average age of onset was 12.55 ± 1.57 years. A total of 44 cSLE without LMV were included in the non-LMV group, and the average age of onset was 12.27 ± 1.60 years. The ratio of male-to-female was 2:9 in both groups. The average course of the disease at onset was 1.95 ± 2.42 months in the LMV group, and the average course of the disease at onset was 3.93 ± 8.03 months in the non-LMV group, but there was no statistically significant difference between the two groups (*t* =  − 0.728, *P* = 0.470). The mean SLEDAI-2 K score at onset was 19.45 ± 10.10 points in the LMV group, while the mean SLEDAI-2 K score of the non-LMV group was 15.93 ± 6.88 points, but there was no statistically significant difference between the two groups (*t* = 1.377, *P* = 0.174).

### Clinical manifestations

**Fig. 1:**
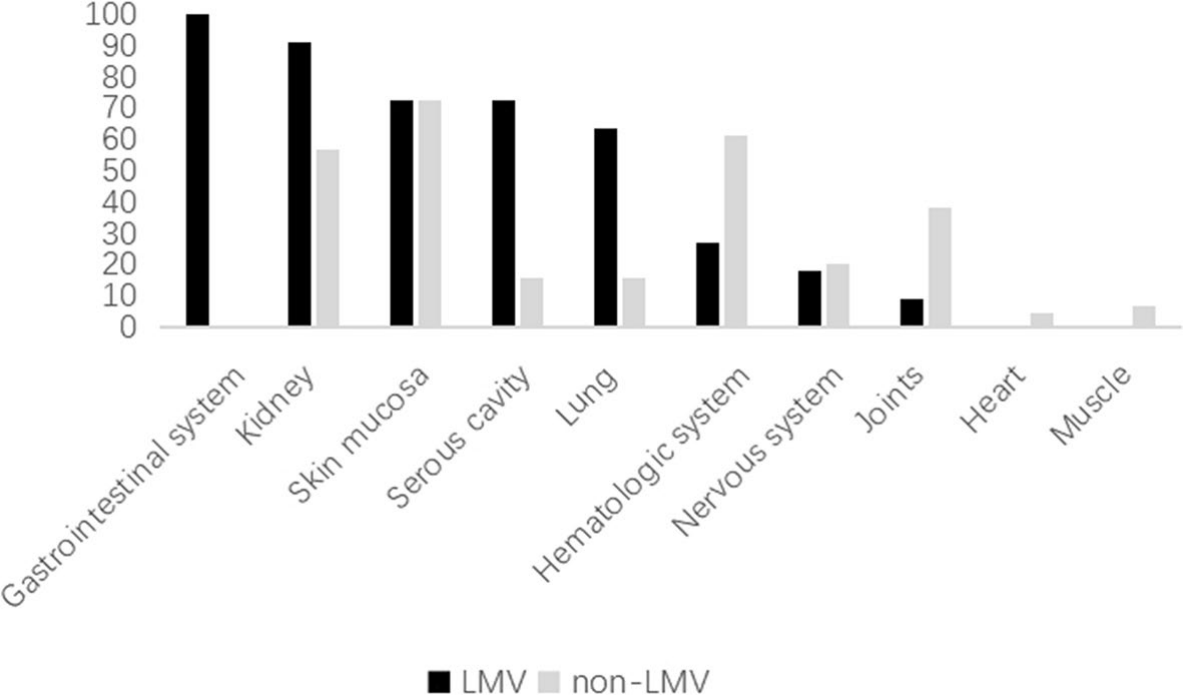
Percentage of organs damaged in the LMV group and the non-LMV group (%)

In the LMV group, 11 cases had abdominal pain (100.0%), 8 cases had vomiting (72.7%), 6 cases had abdominal distension (54.5%), 5 cases had diarrhea (45.5%), and there were no other gastrointestinal manifestations such as acid reflux, belching, and bloody stool. In addition, 8 cases had a malar rash (72.7%), 4 cases had a fever (36.4%), 3 cases had a recurrent oral ulcer (27.3%), and 1 case had alopecia and joint pain (9.1%). Compared with the non-LMV group, the percentage of patients having abdominal pain, vomiting, abdominal distension, and diarrhea was higher in the LMV group (*P* < 0.05). There was no significant difference between the two groups in other clinical manifestations (Table 1). Regarding organ damage, 11 cases had the gastrointestinal system involved (100%); 10 cases had the kidney involved (90.9%), 8 cases had skin and mucosa involved (72.7%), 8 cases had the serous cavity involved (72.7%), 7 cases had the lung involved (63.6%), 3 cases had the hematologic system involved (27.3%), 2 cases had the nervous system involved (18.2%), 1 case had joint involved (9.1%), and 0 cases had the heart and muscle involved. Compared with the non-LMV group, the percentage of the gastrointestinal system (${\chi }_{{\text{C}}}^{2}$ = 48.928, *P* = 0.000), serous cavity (${\chi }_{{\text{C}}}^{2}$ = 11.602, *P* = 0.001), kidney (${\chi }_{{\text{C}}}^{2}$ = 3.069, *P* = 0.042), and lung damage (${\chi }_{{\text{C}}}^{2}$ = 2.938, *P* = 0.049) were higher in the LMV group. There was no statistical difference in other organs damaged (Fig. 1).

**Table 1:** Main clinical manifestations in the LMV group and the non-LMV group (%)

**Clinical manifestations**	**LMV group (*n***_**1**_** = 11)**	**Non-LMV group (*n***_**0**_** = 44)**	${{\varvec{\chi}}}_{\mathbf{C}}^{2}$	***P***
Abdominal pain	11 (100.0)	2 (4.5)	38.507	**0.000**
Vomiting	8 (72.7)	3 (6.8)	19.950	**0.000**
Abdominal distension	6 (54.5)	0 (0.0)	21.619	**0.000**
Diarrhea	5 (45.5)	2 (4.5)	9.832	**0.002**
Malar rash	8 (72.7)	34 (77.3)	0.006	0.937
Fever	4 (36.4)	32 (72.7)	3.664	0.056
Recurrent oral ulcer	3 (27.3)	10 (22.7)	0.006	0.937
Alopecia	1 (9.1)	13 (29.5)	1.012	0.314
Joint pain	1 (9.1)	17 (38.6)	2.276	0.131

### Laboratory findings

The laboratory findings of the two groups at onset are shown in Table 2. Compared with the non-LMV group, the hemoglobin and urea nitrogen in the LMV group were higher (*P* < 0.05), and the difference in other laboratory findings was not statistically significant. There was no statistically significant difference in autoantibody positivity between the two groups (Table 3).

**Table 2:** The laboratory findings in the LMV group and the non-LMV group

Laboratory findings	LMV group (*n*_1_ = 11)	Non-LMV group (*n*_0_ = 44)	*t/t*	*P*
WBC (× 10^9^/L)	6.70 ± 5.03	5.44 ± 3.93	0.896	0.374
HGB (g/L)	118.73 ± 20.51	104.09 ± 19.09	2.242	0.029^**^
PLT (× 10^9^/L)	236.00 ± 119.47^a^	200.89 ± 123.69	0.815	0.419
ALT (U/L)	32.60 ± 23.18^a^	47.47 ± 52.90	− 0.724	0.472
AST (U/L)	41.31 ± 25.19^a^	81.64 ± 151.53	− 0.745	0.460
TBIL (μmol/L)	11.89 ± 9.52^a^	15.17 ± 42.39^a^	− 0.216	0.830
DBIL (μmol/L)	4.60 ± 5.13^a^	7.05 ± 25.31^a^	− 0.270	0.788
Cr (μmol/L)	81.23 ± 78.18^a^	49.32 ± 25.85^a^	1.211^b^	0.259
BUN (mmol/L)	8.64 ± 9.33^a^	5.17 ± 3.04^a^	1.103^b^	0.048^**^
CCR (ml/m^2^)	111.36 ± 68.96^a^	133.57 ± 58.54^a^	− 1.042	0.302
C4 (g/L)	0.06 ± 0.05^a^	0.08 ± 0.09^a^	− 0.721	0.474
24-h urine protein quantification (mg/d)	1221.55 ± 1216.45	960.76 ± 1676.17^a^	0.483	0.631

^**^The difference between the two groups was statistically significant (*P* < 0.05)

^a^Some data is missing

^b^The overall variance is not uniform; the *t* test is adopted

**Table 3:** The positive rate of autoantibodies between the LMV group and the non-LMV group (%)

Autoantibodies	LMV group (*n*_1_ = 11)	Non-LMV group (*n*_0_ = 44)	${\chi }_{{\text{C}}}^{2}$	*P*
dsDNA	8 (72.7)	31 (70.5)	0.050	0.824
SSA	7 (63.6)	17 (38.6)	1.3355	0.248
RO-52	6 (54.5)	13 (29.5)	1.452	0.228
Nucleosome	3 (27.3)	23 (52.3)	1.138	0.252
SSB	2 (18.2)	5 (11.4)	0.010	0.919
Ribosomal	2 (18.2)	19 (43.2)	1.391	0.238
Sm	2 (18.2)	16 (36.4)	0.625	0.429
RNP	2 (18.2)	18 (40.9)	1.105	0.293
Histone	1 (9.1)	18 (40.9)	2.659	0.103
Mitochondrial	1 (9.1)	2 (4.5)	2.207	1.137
ANCA	0 (0.0)	2 (5.1)^a^	―^b^	1.000
ACL-IgG	2 (18.2)	7 (15.9)	0.075	0.785
ACL-IgM	1 (9.1)	6 (13.6)	0.010	0.919
β2GPI	1 (9.1)	12 (27.3)	0.762	0.383

^a^Some data is missing

^b^Fisher’s exact inspection

### Imaging

#### Abdominal-enhanced Computed Tomography (CT)

**Fig. 2:**
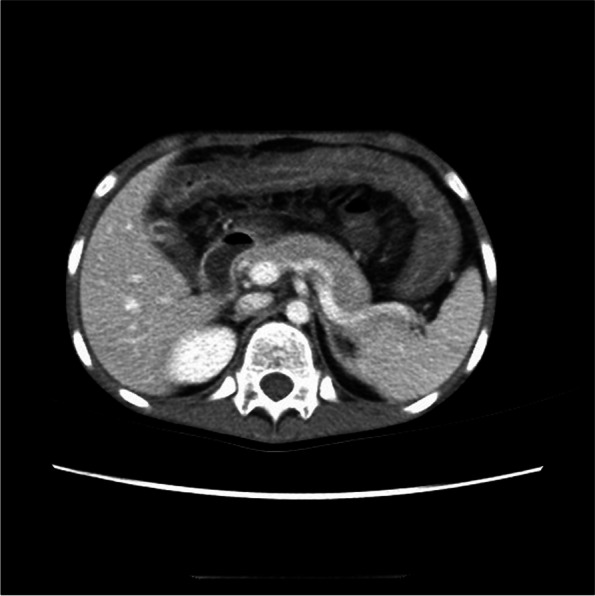
Intestinal wall thickening in LMV case. The mucosa, muscularis propria, and serosa of the intestinal wall were significantly enhanced, and the submucosa edema was significant in abdominal-enhanced CT

**Fig. 3:**
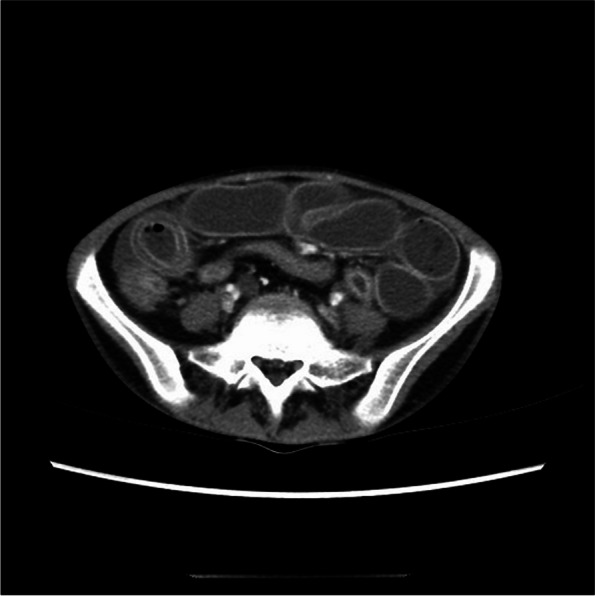
“Double-ring” or “target” sign in the LMV case. The thickening of the intestinal tube wall showed a double-ring or target sign, with obvious enhancement in the inner and outer layers of the mucosa, muscularis propria, and serosa, respectively. The middle layer was characterized by submucosa edema, with no obvious enhancement. Abdominal effusion could be seen in the intestinal space

**Fig. 4:**
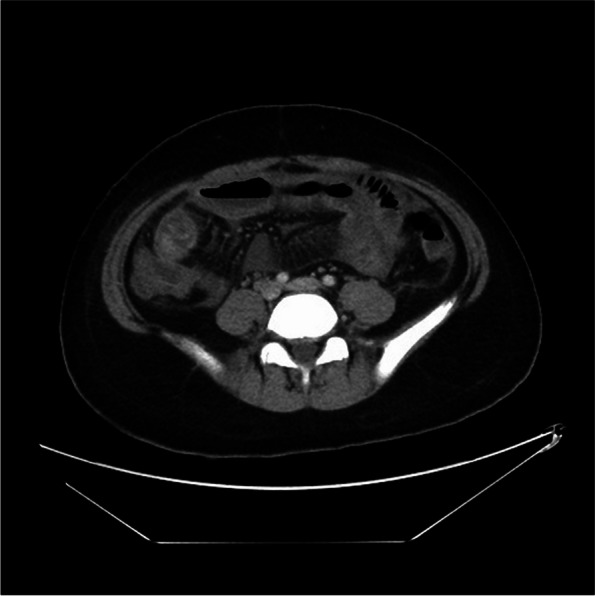
“Comb” sign in the LMV case. The number and expansion of the straight arterioles in the mesentery of the ileum increase, and the distance between them increases, showing the comb sign

In the LMV group, all 11 cases did abdominal-enhanced CT, which showed that the mucosa, muscularis propria, and serosa of the intestinal wall were significantly enhanced, and the submucosa edema was significant (Fig. 2). Intestinal wall thickening could be characterized by double-ring or target sign in CT (Fig. 3). Vessels in mesentery increased, the distance between vessels expanded, so the enhanced CT showed mesenteric vascular enhancement, and the comb sign appeared (Fig. 4). In the non-LMV group, 17 patients did abdominal-enhanced CT. The percentage of intestinal wall thickening, peritoneal effusion, mesenteric vascular enhancement, hydronephrosis with ureteral dilatation, intestinal congestion, and gastric mucosa thickening in the LMV group were higher than those in the non-LMV group (*P* < 0.05), while the splenomegaly was lower (*P* < 0.05) (Table 4).

**Table 4:** Imaging of abdominal-enhanced CT in the LMV group and the non-LMV group (%)

Imaging	LMV group (*n*_1_ = 11)	Non-LMV group (*n*_0_ = 17)	P^*^
Intestinal wall thickening	11 (100.0)	0 (0.0)	0.000^**^
Peritoneal effusion	9 (81.8)	2 (11.8)	0.000^**^
Mesenteric vascular enhancement	6 (54.6)	0 (0.0)	0.001^**^
Hydronephrosis with ureteral dilatation	5 (45.5)	0 (0.0)	0.005^**^
Intestinal congestion and liquid–gas level	3 (27.3)	0 (0.0)	0.005^**^
Gastric mucosa thickening	3 (27.3)	0 (0.0)	0.005^**^
Thickening of the bladder wall	1 (9.1)	0 (0.0)	0.393
Splenomegaly	1 (9.1)	8 (47.1)	0.049^**^
Fatty liver	1 (9.1)	1 (5.9)	1.000
Hepatomegaly	0 (0.0)	6 (35.3)	0.055
Splenic infarction	0 (0.0)	1 (5.9)	1.000
Pancreatitis	0 (0.0)	1 (5.9)	1.000

^*^Fisher’s exact inspection

^*^^*^The difference between the two groups was statistically significant (*P* < 0.05)

#### The gastrointestinal ultrasound

In the LMV group, 11 cases underwent this examination: 6 cases had intestinal wall thickening (54.55%), 6 cases had peritoneal effusion (54.55%), 4 cases had gastric wall thickening (36.36%), 2 cases had hydronephrosis with ureteral dilatation (18.18%), 2 cases had mesenteric edema and thickening (18.18%), and 1 case had enlarged mesenteric lymph nodes, intestinal dilatation, and bile salt deposition (9.09%). In the non-LMV group, 28 underwent this examination, and only 2 cases had peritoneal effusion (7.14%), while other gastrointestinal abnormalities were not seen.

#### Abdominal plain

**Fig. 5:**
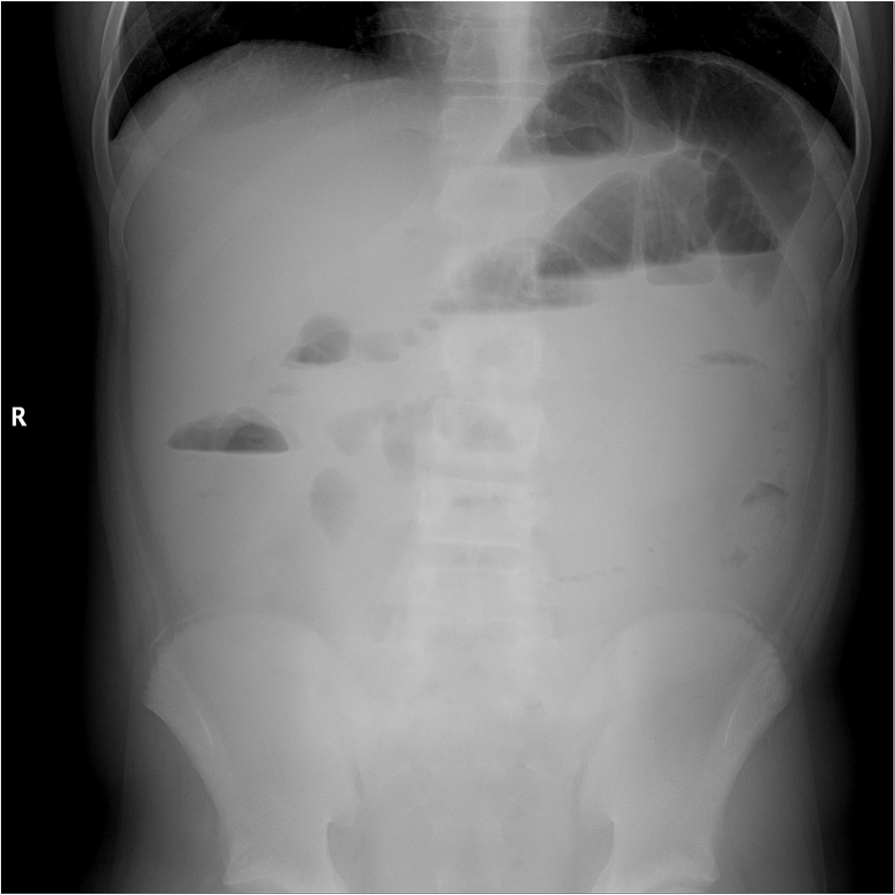
Abdominal plain in LMV case. Intestinal congestion and liquid–gas level, suggesting intestinal obstruction

In the LMV group, 3 patients underwent abdominal plain. These 3 cases had intestinal congestion and liquid–gas level (100%), which suggested intestinal obstruction (Fig. 5). No case in the non-LMV group underwent this examination.

#### Gastroscopy and colonoscopy

In the LMV group, only 1 case underwent gastroscopy and colonoscopy: chronic superficial gastritis, duodenitis, white spots in the duodenum, colitis, and proctitis. One case only underwent gastroscopy: superficial gastritis. No case in the non-LMV group underwent gastroscopy or colonoscopy.

### Treatment

In the LMV group, 10 cases received methylprednisolone (MP) 10–30 mg/kg/d pulse therapy (90.9%), followed by prednisolone 1–2 mg/kg/day; only one case just received prednisone 1–2 mg/kg/day (9.1%). All 11 cases were treated by cyclophosphamide (CTX) 1 g/m^2^ pulse therapy (100%) once a month for 6 months, and then once every 3 months, 3 more times. Nine cases were treated with hydroxychloroquine (81.8%). Three cases had belimumab as the initial treatment, one case was treated by belimumab at the second month after the initial treatment, and one child was treated with rituximab at the third month. No case underwent surgical treatment.

Compared with the non-LMV group, the percentage of patients receiving MP pulse therapy (${\chi }_{{\text{C}}}^{2}$ = 3.510, *P* = 0.037) and CTX pulse therapy (${\chi }_{{\text{C}}}^{2}$ = 4.016, *P* = 0.023) was higher in the LMV group. But the percentage of patients receiving prednisone or MP 1–2 mg/kg/day treatment (${\chi }_{{\text{C}}}^{2}$ = 3.510, *P* = 0.037) and hydroxychloroquine treatment (${\chi }_{{\text{C}}}^{2}$ = 3.924, *P* = 0.037) was lower than that in the non-LMV group.

### Prognosis

In the LMV group, 10 cases had complete follow-up data. The average follow-up time was 23.40 ± 26.22 months, with no death. The follow-up materials are shown in Table 5. In the non-LMV group, 36 had complete follow-up data, and the average follow-up time was 16.44 ± 10.55 months, with no death. Comparing the SLEDAI-2 K score, 24-h urinary protein, complement 3 (C3), and complement 4 (C4) between the two groups after 1, 3, 6, and 12 months, we found that the 24-h urinary protein at 3 and 6 months in the LMV group was higher (*t* = 2.635, *P* = 0.013; *t* = 2.371, *P* = 0.025), and the C3 was lower than that in the non-LMV group at 6 and 12 months (*t* =  − 2.094, *P* = 0.043; *t* =  − 2.067, *P* = 0.049). After 12 months of treatment, the SLEDAI-2 K score in the LMV group was higher than that in the non-LMV group (*t* = 2.643, *P* = 0.014).

**Table 5:** Follow-up materials in the LMV group

The follow-up materials	1 month after treatment (*n*_1_ = 10)	3 months after treatment (*n*_2_ = 9)	6 months after treatment (*n*_3_ = 8)	12 months after treatment (*n*_4_ = 4)
Clinical manifestations	None	1 case had vomiting again	2 cases had new rash	2 cases had vomiting and diarrhea again
WBC (× 10^9^/L)	8.73 ± 3.35	7.71 ± 2.88	5.55 ± 2.12	4.35 ± 3.38
HGB (g/L)	125.10 ± 15.13	135.50 ± 22.25	129.38 ± 16.28	128.4 ± 12.97
PLT (× 10^9^/L)	212.40 ± 19.83	238.13 ± 41.73	255.00 ± 34.57	253.00 ± 54.09
C3 (g/L)	0.74 ± 0.24	0.89 ± 0.17	0.82 ± 0.13	0.68 ± 0.23
C4 (g/L)	0.12 ± 0.05	0.16 ± 0.06	0.15 ± 0.05	0.12 ± 0.09
24-h urine protein quantification (mg/d)	151.13 ± 109.54	1484.94 ± 2322.15	1511.54 ± 1814.48	801.98 ± 1069.25
SLEDAI-2 K	-	2.25 ± 1.25	4.50 ± 3.93	8.00 ± 5.34
The positive rate of anti-dsDNA antibody (%)	71.4	37.5	50.0	75.0
Abdominal-enhanced CT	Normal (3 cases)	Normal (2 cases)	Normal (2 cases)	1 case: normal 2 cases: intestinal wall thickening

## Discussion

We found that LMV often occurs in 12 ~ 13-year-old girls with high disease activity of cSLE, with an acute onset, requiring clinicians to identify it as soon as possible. LMV is a critical condition of SLE, and the case-fatality rate is 50% [4]. The main pathological changes are arteritis and phlebitis with fibrin necrosis, fibrin thrombosis, and inflammatory cell infiltration [1, 5]. There are few reports of LMV in cSLE, and the LMV at onset is even rarer. In 2017, we reported three cSLE with LMV at onset [6], which are included in this study. We found that cSLE with LMV as initial presentation often occurs in 12 ~ 13-year-old girls. The average course of onset was 1.95 months, which was shorter than that in all cSLE in adolescence by 10 months [7]. The SLEDAI-2 K score at onset was 19.45 points, indicating disease activity was very high. In 2021, Wang et al. compared adult SLE with and without LMV and found that SLEDAI-2 K in patients with LMV was higher than that in patients without LMV [8]. In 2011, Tu et al. compared the clinical characteristics of adult LMV and child LMV, and the study showed that the SLEDAI-2 K score of child LMV was higher than that of adult LMV patients, with a score of > 15 points [9].

In our study, all LMV had abdominal pain at onset (100%), with varying degrees, but it was rare in cSLE without LMV. According to the literature, the most common clinical manifestations of LMV are abdominal pain, nausea, vomiting, diarrhea, black stool, hematemesis, abdominal distension, etc. [2, 4, 5, 10]. Therefore, cSLE with acute abdominal pain need to be alert to LMV. Our study also found other gastrointestinal symptoms had some clinical value in identifying LMV. At the same time, LMV patients are more susceptible to damage to the kidney, serous cavity, and lung, so clinicians need to pay attention to these organs. Renal involvement was one of the top causes of death in SLE [11], and cSLE had a significantly higher incidence of renal involvement than adult SLE [12]. In our study, 10 LMV cases had kidney involvement, with an average 24-h urine protein of 1221.55 mg, and BUN was higher than that of children without LMV. We also found that cSLE with LMV often had decreased complement C3 and C4. There are similar findings in a paper: complement C3 and C4 are lower in LMV patients than in other SLE [8]. Therefore, clinicians should pay close attention to abdominal symptoms in cSLE. Once LMV is considered, it is necessary to focus on the kidney, serous cavities, and lung.

Currently, the gold standard for diagnosing LMV is abdominal-enhanced CT. The most typical manifestations of LMV are intestinal wall thickening, “target sign,” segmental intestinal dilatation, mesenteric vascular enhancement, mesenteric fat blurring, and peritoneal effusion [4]. In our study, comparing with cSLE without LMV, the proportion of intestinal wall thickening, peritoneal effusion, mesenteric vascular enhancement, hydronephrosis with ureteral dilation, intestinal congestion, and gastric mucosal thickening in cSLE with LMV were increased. Among them, intestinal wall thickening was the most common, accounting for 95% of LMV according to literature reports [13]. In our study, all cSLE with LMV presented with intestinal wall thickening. The main pathological changes are intestinal wall edema and vascular inflammation, so a typical target sign or double loop sign can be presented on enhanced CT. Mesenteric vascular enhancement occurred in 6 cases in our study, presenting as a comb tooth sign, which is also a specific imaging manifestation in LMV [8]. We had 3 cases with intestinal congestion with liquid–gas level and diagnosed as intestinal obstruction, which improved after glucocorticoid treatment. Therefore, they were considered pseudointestinal obstruction. Pseudointestinal obstruction caused by lupus is rare, accounting for approximately 5% of abdominal pain in SLE [14], and cSLE is even rarer. The pathological mechanism is not clear. Smooth muscle and vasculitis-related autoantibodies may be one of the reasons [10]. It is effective for high-dose glucocorticoid therapy [14]. Four LMV had bilateral hydronephrosis and bilateral ureteral dilatation in our study. In 2021, Wang et al. found that LMV with hydronephrosis was 62.5%, and urinary tract dilatation was 50% [8], the cause of which may be vascular inflammation leading to smooth muscle involvement [15]. Gastrointestinal ultrasound is widely used in pediatrics with no radiation and low cost. This time, we also analyzed the gastrointestinal ultrasound in LMV. Compared with abdominal-enhanced CT, gastric wall thickening is more sensitive in ultrasound, and other imaging signs are less sensitive than CT. So gastrointestinal ultrasound could be used as a preliminary method for LMV. If LMV is highly suspected, abdominal-enhanced CT should be performed as soon as possible.

Because LMV is a critical condition of cSLE, sufficient treatment is needed. According to the literature, MP pulse therapy combined with CTX pulse therapy was the fastest and most effective treatment for LMV. After that, maintenance treatment with prednisone could avoid intestinal necrosis and perforation [5, 16]. In our study, 10 of the 11 cSLE with LMV received MP pulse combined with CTX pulse therapy, and the proportion of using this treatment was higher than that without LMV. After treatment, the symptoms relieved quickly, and no complications such as intestinal necrosis and perforation occurred. Recently, we tried to use belizumab to treat severe active cSLE with LMV, and the efficacy is satisfied, but we need more data to verify.

In our study, the clinical symptoms of LMV disappeared quickly after 1 month. Only one case experienced gastrointestinal symptoms again after 2 months, and at 12 months, two cases experienced gastrointestinal symptoms again. One month after initial treatment, the 24-h urine protein was basically returned to normal, and C3 and C4 increased, especially C4, which had returned to normal. The abdominal imaging data were incomplete. After 3 months, the average SLEDAI-2 K score was 2.25 points, indicating that the disease was inactive. During the entire follow-up process, there were no deaths. Compared with cSLE without LMV, LMV had higher 24-h urinary protein, lower complement C3, and higher SLEDAI-2 K scores. We found that after sufficient treatment, cSLE with LMV had a rapid disease recovery, low relapse, and a good prognosis, but the overall recovery is worse than without LMV. Because the sample size was limited in our study, it is necessary to further study to confirm.

## Conclusions

LMV often occurs in 12 ~ 13-year-old girls with high disease activity of cSLE, with an acute onset. Abdominal pain is the most common clinical manifestation and more susceptible to damage to the kidney, serous cavity, and lung in cSLE with LMV. Abdominal-enhanced CT should be performed as soon as possible in suspected cases. Methylprednisolone pulse combined with CTX pulse therapy is effective. After the treatment above, cSLE with LMV has a good prognosis, but the overall recovery is worse than non-LMV; we still need further confirmation.

## Data Availability

The data used in this study are available from the first or corresponding author upon reasonable request.

## Abbreviations

cSLE: Childhood-onset systemic lupus erythematosus
SLE: Systemic lupus erythematosus
LMV: Lupus mesenteric vasculitis
EULAR: European League Against Rheumatology
ACR: American College of Rheumatology
SLEDAI-2K: Systemic Lupus Erythematosus Disease Activity Index-2000
CT: Computed tomography
MP: Methylprednisolone
CTX: Cyclophosphamide
C3: Complement 3
C4: Complement 4
